# Insights into the Correlation between Microbial Community Succession and Pericarp Degradation during Pepper (*Piper nigrum* L.) Peeling Process via Retting

**DOI:** 10.3390/foods13111615

**Published:** 2024-05-23

**Authors:** Yuting Fu, Shuai Chen, Xinjun Wang, Lu Wang, Zexin Wang, Yanfei Cheng, Yuyi Liu, Lin Zhang, Sixin Liu, Jiamu Kang, Congfa Li

**Affiliations:** 1 School of Food Science and Engineering, Hainan University, Haikou 570228, China; 21210832000004@hainanu.edu.cn (Y.F.); wangxinjuner@163.com (X.W.); 22220951350082@hainanu.edu.cn (Y.L.);; 2 Key Laboratory of Food Nutrition and Functional Food of Hainan Province, Haikou 570228, China; 3 Key Laboratory of Tropical Agricultural Products Processing Technology of Haikou, Haikou 570228, China

**Keywords:** pepper peeling, retting, microbial community, metabolite profile, pectin degradation

## Abstract

White pepper, used both as a seasoning in people’s daily diets and as a medicinal herb, is typically produced by removing the pericarp of green pepper through the retting process. However, the mechanism of the retting process for peeling remains unclear. Therefore, this study aimed to investigate the changes in physicochemical factors, microbial community succession effects, and metabolites of the pepper pericarp during the pepper peeling process. The findings indicated that pre-treatment involving physical friction before the retting process effectively reduced the production time of white pepper. During the retting process, the pectinase activity increased, leading to a decrease in the pectin content in the pepper pericarp. There was a significant correlation observed between the changes in pH, pectin content, and peeling rate and the Shannon diversity index of bacteria and fungi. *Prevotella*, *Lactococcus*, and *Candida* were the dominant microbial genera during the retting. The functional predictions suggested that the monosaccharides degraded from the pepper pericarp could have been utilized by microbes through sugar metabolism pathways. Metabolomic analysis showed that the metabolic pathways of carbohydrates and amino acids were the main pathways altered during the pepper peeling process. The verification experiment demonstrated that the degradation of pectin into galacturonic acid by polygalacturonase was identified as the key enzyme in shortening the pepper peeling time. The structure of the pepper pericarp collapsed after losing the support of pectin, as revealed by scanning electron microscopy. These results suggest that the decomposition of the pepper pericarp was driven by key microbiota. The succession of microbial communities was influenced by the metabolites of the pepper pericarp during retting. These findings provide new insights into the retting process and serve as an important reference for the industrial production of white pepper.

## 1. Introduction

Pepper products, including green pepper, black pepper, and white pepper [1], are well-known for their distinct uses and qualities. White pepper is not only used in cooking but also for medicinal purposes [2]. Its mild, spicy taste makes it a popular choice for enhancing the flavor of dishes [3]. Moreover, pepper has been reported to offer numerous health benefits, including promoting digestion, anti-inflammatory effects, and antioxidant properties, thereby holding high commercial value [4].

Various methods are used to process green pepper into white pepper, including chemical methods, physical methods, and biological methods [5]. The chemical peeling method effectively removes the pepper pericarp but can result in chemical residues on the peppers, posing a risk to consumer health [6]. The mechanical peeling method improves efficiency but may damage the appearance, impacting the quality and commercial value of the product [7]. Biological methods involve specific microorganisms that produce enzymes to achieve efficient pepper peeling [8]. However, the commercial application of enzymes sourced from microorganisms is limited due to their high cost [9]. The conventional peeling process usually involves the retting of peppers for 7 to 15 days [10]. The extended soaking time required by this method has been found to generate an unpleasant flavor and compromise the quality of white pepper. Moreover, the soaking water tends to become foul-smelling, thereby causing environmental pollution.

Therefore, the aim of this study was to use a method that combines mechanical friction and retting for pretreatment. The potential mechanism may involve the degradation of the pericarp and the interactions between the microbial community and key components of the pericarp during retting. To comprehensively understand this process, high-throughput sequencing was used to analyze the succession of microbial communities during the degradation of pepper pericarp. The changes in metabolite profiles during the retting process were analyzed using untargeted metabolomics. This study will provide a new method to shorten the production time of white pepper while reducing its environmental effects.

## 2. Materials and Methods

### 2.1. Experimental Design and Sample Collection

Fresh green peppers were harvested from a pepper farm in Hainan Province, China. Prior to the retting process, the pepper fruits were subjected to a physical friction pretreatment in the peeling machine for 3 min (model D-300, Dezhou Hongkang Machinery Equipment Co., Ltd., Dezhou, China). Subsequently, the peppers were retted at room temperature in a citric acid buffer solution with a pH of 5.5 at a 1:4 ratio (fruit to solution). Samples of pepper pericarp and soaking solution were collected at various time points throughout the retting process (0 h, 12 h, 24 h, 36 h, 48 h, and 60 h). At each time point, three parallel samples were collected, as follows: A 90-mL sample of the soaking solution was collected and filtered, followed by centrifugation for 5 min (12,000× *g*, 4 °C). Then, the supernatant and precipitate were collected and stored at −80 °C until use. Also, the pepper pericarp was collected; a portion was directly stored at 4 °C for subsequent use, while the remaining part was freeze-dried and stored at −80 °C for metabolomic analysis.

### 2.2. Measurement of Physicochemical Properties

The pH of the supernatant was measured using a PHS-3C pH meter (OUSTOR, Shanghai, China). The peeling rate of pepper is expressed as (m1 − m2)/m1, where m1 is the fruit weight of pepper before peeling (g) and m2 is the fruit weight of pepper after peeling (g). The composition of the pericarp, including pectin, cellulose, and hemicellulose, was measured and expressed in mg/g of pericarp [11]. The quantification of the composition in pepper pericarp and the measurement of enzymatic activity were conducted utilizing reagent kits supplied by Suzhou Geruisi Biotechnology Co., Ltd. (Suzhou, China). The activity of pectinase was defined as one unit (U), corresponding to the production of 1 mg of galacturonic acid from the sample per gram per hour. After homogenizing 1 g of pericarp with 1 mL of ethanol in an ice bath, the pericarp was placed at 4 °C for 5 min. The samples were then centrifuged for 5 min (12,000× *g*, 4 °C), and the supernatant was removed. Following the repetition of this step twice, 1 mL of extract was introduced into the precipitate, followed by vortex mixing. After standing, the mixture was centrifuged, and the precipitate was then discarded [12]. The supernatant was added to the reagent according to the instructions of the kit and then tested. Reagents used in pectinase activities were from commercial test kits (Suzhou Grace Biotechnology Co., Ltd., Suzhou, China) based on the manufacturer’s recommendations.

### 2.3. Extraction and Sequencing Analysis of Microbial DNA

The total DNA of each collected sample was extracted using an E.Z.N.A. Soil DNA Kit (Omega Bio-Tek, Norcross, GA, USA), according to the manufacturer’s instructions. The DNA concentration and quality were measured by a NanoDrop spectrophotometer. Then, PCR amplification was performed using specific primer pairs: 27F (AGAGTTTGATCCTGGCTCAG) and 338R (TGCTGCCTCCCGTAGGAGT) for the 16S rRNA gene V1-V2 region of the bacteria, and ITS1F (CTTGGTCATTTAGAGGAAGTAA) and ITS2R (GCTGCGTTCTTCATCGATGC) for the ITS region of the fungi, using an ABI GeneAmp^®^ 9700 PCR system and TransStart^®^ FastPfu DNA Polymerase. Following amplification, the PCR products were quantified using the QuantiFluor™—ST Blue Fluorescence Quantitation System and then prepared for sequencing (Promega Corporation, Madison, WI, USA) [13].

DNA fragments were recovered using the AxyPrep DNA Gel Extraction Kit (Axygen Biosciences, New York, NY, USA), eluted with Tris HCl, and stored at −20 °C, followed by separation using 2% agarose gel electrophoresis. The quantity of these fragments was measured using a QuantiFluor™ (Promega Corporation, Madison, WI, USA). Libraries were then constructed using the TruSeqTM DNA Sample Prep Kit and sequenced on the Illumina NovaSeq platform (Illumina, San Diego, CA, USA). The original data were filtered and spliced using Flash (v 1.2.11) software, and operational taxonomic unit (OTU) clustering and taxonomic analysis were performed using Uparse11 software based on the Silva (Release 138) bacterial database and Unite (Release 8.0) fungal database, based on RDP Classifier (v 2.13) [14].

### 2.4. Metabolite Extraction and LC-MS Analysis

A 50 mg freeze-dried pepper pericarp sample was processed by placing it in a 2 mL centrifuge tube with a 6 mm grinding bead, and metabolites were extracted using 400 μL of a methanol: water 4:1 (*v*/*v*) solution with 0.02 mg/mL L-2-chlorophenylalanine. The sample was then ground for 6 min at −10 °C and 50 Hz using a frozen tissue grinder (Shanghai wanbo biotechnology Co., Ltd., Shanghai, China), followed by low-temperature ultrasonic extraction for 30 min at 5 °C and 40 kHz. After a 30 min rest at −20 °C, the sample was centrifuged at 4 °C and 13,000× *g* for 15 min, with the supernatant then transferred to a sample vial for liquid chromatography-mass spectrometry (LC-MS) analysis. LC-MS conditions involved chromatographic separation on an HSS T3 column with 95% water and a mix of acetonitrile, isopropanol, and water, with a gradient increasing from 0% to 100%, a flow rate of 0.40 mL/min, and a column temperature of 40 °C. Mass spectrometry was conducted in positive (3500 V) and negative (2800 V) ion modes, scanning from *m*/*z* 70 to 1050 with a sheath gas pressure of 40 psi and an ion source temperature of 400 °C [15]. Data were processed using Progenesis QI software (version 1.9.1, Waters Corporation, Milford, CT, USA), involving baseline filtering, peak identification, and referencing HMDB (http://www.hmdb.ca/, version 5.0, accessed on 10 November 2023) and Metlin (https://metlin.scripps.edu/, accessed on 10 November 2023).

### 2.5. Enzyme-Based Pericarp Removal Validation Experiment

The pre-treatment steps for green pepper were the same as in 2.1. According to the results of previous experiments, 1% of the commercial enzyme was added to the soaking solution. The treated green peppers were soaked in a solution containing these enzymes. Samples were taken every 2 h to observe and calculate the degree of peeling to determine the peeling rate, continuing this process until the green peppers were completely peeled. The pure enzyme preparations contain pectinase (FDY-2212), cellulase (FDY-2243), hemicellulase (FDG-2255), pectin lyase (FDY-2211) and pectinesterase (FYD-2272), purchased from SUNSON Enzyme Biotechnology Co., Ltd., and polygalacturonase (P299306), obtained from Shanghai Aladdin Bio-Chem Technology Co., Ltd. (Shanghai, China). Pepper fruits at 0 h, 4 h, and 8 h were fixed in a 2.5% glutaraldehyde (G1102, Servicebio, Wuhan, China) solution. Following ethanol dehydration, the pepper fruits were subjected to drying using a critical point drying method. Samples were mounted on metallic stubs using carbon stickers and sputter-coated with gold for 30 s. Scanning electron microscopy (SU8100, HITACHI, Tokyo, Japan) was used for observing the structure of the pepper pericarp.

### 2.6. Bioinformatic and Statistical Analyses

SPSSPRO (https://www.spsspro.com/, accessed on 23 October 2023) was used for one-way ANOVA to analyze the significance of physicochemical properties, employing LSD for post hoc comparisons. Experimental results were expressed as mean values ± standard deviation from three replicates. Statistical significance was determined by a significance level of *p* < 0.05 for assessing differences between the groups.

The Chao1 and Shannon indices were calculated by using Mothur (https://www.mothur.org/wiki/Download_mothur, version 1.30.2, accessed on 15 November 2023). Community bar charts and principal component analysis (PCA) were conducted using R software (version 3.3.1). Discriminant analysis effect size (LEfSe) along with linear discriminant analysis (LDA), setting the threshold for LDA scores at >4 and significance at *p* < 0.05, were employed to identify key microbial genera that significantly changed during retting. Correlation network models were visualized using Cytoscape (version 3.7.1). PICRUSt2 (https://github.com/picrust/picrust2/, version 2.2.0, accessed on 8 November 2023) was employed to predict microbial community metabolic pathways.

PCA and correlation heatmaps were constructed using MetaboAnalyst (https://www.metaboanalyst.ca/, accessed on 11 November 2023). Significantly different metabolites were identified with VIP > 1 and *p* < 0.05, based on partial least squares discriminant analysis (PLS-DA) and *t*-test analysis. Identified metabolites were matched against the KEGG Compound database (https://www.kegg.jp/kegg/compound/, accessed on 11 November 2023) to categorize metabolites. These metabolites were mapped into biochemical pathways via KEGG (http://www.genome.jp/kegg/, accessed on 11 November 2023) for metabolic enrichment and pathway analysis [16].

## 3. Results and Discussion

### 3.1. Dynamic Changes of Physicochemical Factors

The peeling rate was considered a key indicator of the efficiency of the pepper peeling process. A long period of retting led to poor-quality pepper, characterized by an abnormal odor and a darker color. [10]. In this study, a new peeling process involving soaking after mechanical friction was introduced. The high efficiency of this process increased the potential value due to the 100% peeling rate within 60 h (Table 1). The pH of the pepper peeling solution was observed to increase from 5.50 to 5.92 and subsequently decrease to 5.50. Wackett et al. found that the pH change might be related to the accumulation of organic acids by the microbial succession [17].

Pectin, cellulose, and hemicellulose, the main components of the pepper pericarp, were identified as crucial for the adhesion of plant cell walls and the maintenance of plant tissue integrity [18]. After 60 h of retting, the pepper’s main component of pectin decreased by 66.38%, cellulose by 43.78%, and hemicellulose by 32.76%. The decreased content of the pericarp indicated that the softening of the pepper pericarp might be related to the increased rate of peeling. During the retting process, the pectinase activity increased significantly from 3.36 mg/h/g to 8.74 mg/h/g. The breakdown of pectin was catalyzed by pectinase, leading to the reduction of pectin in the pepper pericarp. Gebregziabher et al. revealed that the integrity of the pectin structure contributes to the maintenance of the fruit’s hardness. These results showed that the peeling of the pepper pericarp might be related to the decreased content of pectin, which is due to the catalytic action of pectinase [19].

To reveal the relationship between the changes in components of pepper peel and microbial enzyme profiles, we investigated the microbial community structure that may cause these changes, aiming to explore the role of microbiotas and their secreted enzymes in the peeling of pepper during the soaking process.

### 3.2. The Diversity of Microbial Community during the Retting Process

**Figure 1:**
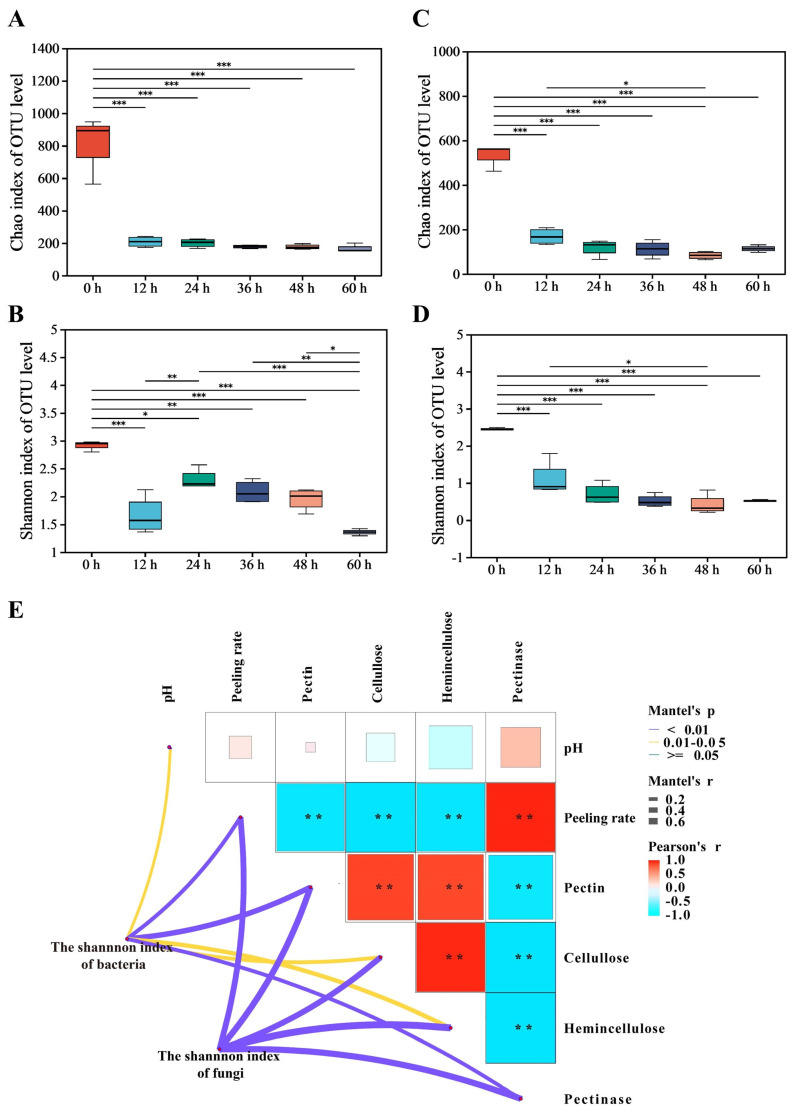
Analysis of microbial community diversity and dissimilarity during the retting process; it includes the bacterial α-diversity indices: Chao1 (**A**), Shannon (**B**); the fungal α-diversity indices: Chao1 (**C**), Shannon (**D**); Mantel test for the correlation between physicochemical factors and microbial communities (**E**); Mantel’s p: Significance level of the result; ** indicates significance 0.01 < *p* < 0.05. Mantel’s r: Strength of correlation between two matrices. Pearson’s r: Correlation strength between physicochemical indexes.

This study investigated changes in microbial richness and diversity during the retting process. When retting began, the Chao1 and Shannon indices for bacteria and fungi in pepper pericarp were the highest, indicating maximal richness and diversity, possibly due to the effect of environmental microorganisms on the richness and diversity (Figure 1A–D). The sequencing depth could cover all microorganisms in the sample, and the rarefaction curves of bacteria and fungi tended to be steady, so the sequencing data volume was reasonable. When retted for 12 h, the decrease in oxygen in the pepper peeling solution affected the growth of aerobic microorganisms and significantly reduced the richness and diversity. The richness and diversity of bacteria and fungi decreased. However, there was a significant difference in the α-diversity index between bacteria and fungi during the retting process, indicating the interactions between microorganisms [20]. When retted for 36 h, the growth of the microbial community became stable. Significant changes in microbial community composition were observed during the retting process.

A Mantel test was conducted to investigate the correlation between the succession of bacterial and fungal communities, represented by the Shannon index, and the physicochemical factors during the retting process (Figure 1E). The succession of the bacterial community showed significant and strong correlations with physicochemical factors such as pectin, pectinase, and peeling rate. Similarly, the succession of the fungal community exhibited significant and strong correlations with pectin, pectinase, cellulose, hemicellulose, and peeling rate. The results suggested that variations in physicochemical factors could be associated with the succession of microbial communities.

### 3.3. Dynamics of the Microbial Diversity during the Retting Process

**Figure 2:**
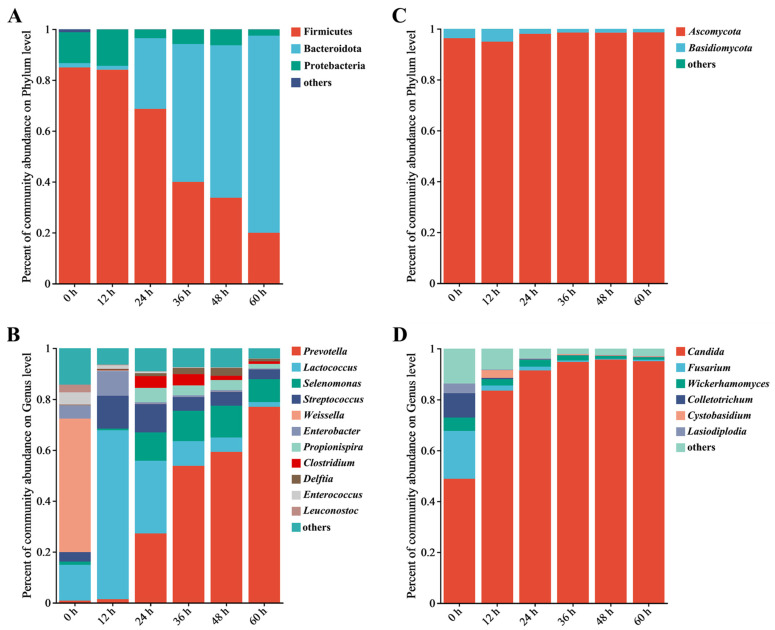
Analysis of the relative abundance of bacteria and fungi during the retting process using bar plots; it includes the bacterial phyla (**A**) and genera (**B**), as well as the fungal phyla (**C**) and genera (**D**). Note: Genera not in the top 10 in abundance are collectively referred to as “other”.

The bacterial community at the phylum level during the retting process is shown in Figure 2A. There was a significant decrease in the abundance of Firmicutes, decreasing from 82.05% in 0 h samples to 21.01% during the retting process. Similarly, Proteobacteria decreased from 15.06% to 2.6%. Actinobacteriota, initially present at 1.16% in 0 h, completely disappeared within 24 h, indicating an inability to withstand the retting water conditions. Figure 2B shows changes in bacterial community composition at the genus level during the retting process. *Prevotella*, *Lactococcus*, *Selenomonas*, *Streptococcus*, *Weissella*, and *Enterobacter* were the dominant genera. These genera were known to degrade plant polysaccharides and used pepper pericarp as a source of nutrition [21]. Notably, the abundance of *Prevotella* increased significantly from 0.87% to 75.45%, becoming dominant with the retting process. *Prevotella*, efficient at degrading hemicellulose and starch, converted hemicellulose into sugars and subsequently into volatile fatty acids [22].

As shown in Figure 2C, Ascomycota has a fungal phyluefficiency of a combined pm, with an abundance of 95.85% in 0 h samples. The abundance reached its peak of 98.36% during the retting process. The abundance of Basidiomycotae decreased from 3.54% to 1.43%. The changes in the fungal communities were not significant throughout the retting process. Other fungal phyla remained consistently below 1%, such as Mucoromycota, Rozellomycota, and Chytridiomycota, during the process. At the genus level (Figure 2D), changes in the fungal community indicated the succession of fungal communities. *Candida* was dominant in the 0 h sample, increasing rapidly from 45% to 82.41% within 12 h, then gradually increasing to about 90% and remaining stable. *Candida* is a widely adaptable hydrolytic enzyme that can effectively use complex polysaccharides [23]. The abundance of *Aspergillus* increased from 0.35% to 2.9% while decreasing due to the reduction of oxygen availability during the retting process [24]. *Candida* was the genus that continued to grow, while other genera decreased. Following the observation of structural changes within bacterial and fungal communities, Bacteroidota and Ascomycota, particularly *Prevotella* and *Candida*, emerged as dominant during the retting process. After observing changes in the structure of the microbial community, we decided to further investigate the key bacteria during the retting process.

### 3.4. Identifying Key Microbes and the Metabolic Function

**Figure 3:**
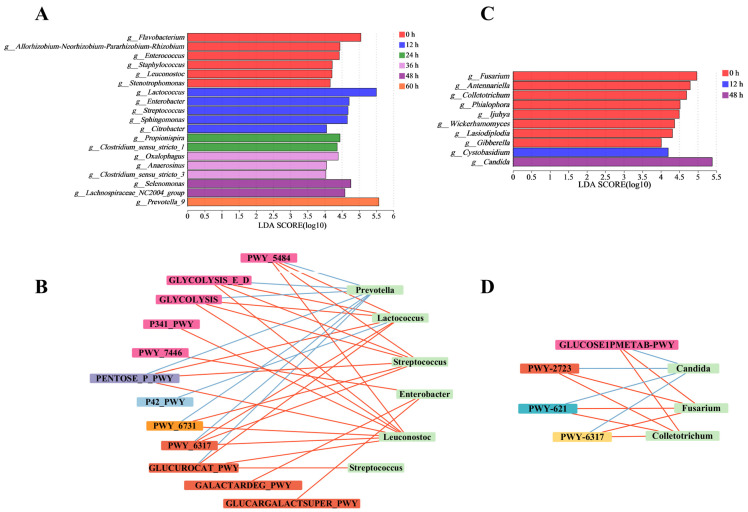
Analysis of significant differences in bacterial and fungal genera during the retting process. Intergroup genera variation analysis for bacteria (**A**) and fungal (**C**). Network correlation analysis of key fungal (**B**) and bacterial (**D**) genera and metabolic pathway.

After analyzing structural changes within bacterial and fungal communities at the phylum and genus levels, we used LEfSe and LDA to identify key microbes. LEfSe enables the identification of microbial communities with statistically significant abundance differences by utilizing LDA to evaluate the impact of these differential microbes [25]. The significant alterations within microbial communities (LDA > 4) indicated their important contribution, enabling us to identify the key microbiota during the retting process. The roles and contributions of bacteria and fungi in the pepper peeling process were different [26]. Notably, bacteria such as *Prevotella*, *Lactococcus*, *Staphylococcus*, *Enterobacter*, and *Leuconostoc* indicated potential connections with the key metabolic pathways, including glycolysis, TCA cycle, pentose phosphate pathway, glycogenesis, glycogenolysis and decomposition of sugars and sugar acids, as depicted in Figure 3A,B. These pathways provided the necessary energy and precursors for pectin-degrading enzymes [27]. Fungi such as *Candida*, *Fusarium*, and *Colletotrichum* are primarily engaged in the decomposition of sugars and sugar acids. Compared to fungi, the metabolic pathways of bacteria are more complex (Figure 3C,D). The sequencing data from key microbial communities was employed for functional analysis. This analysis was accomplished through comparison with the MetaCyc database, aiming to illuminate the metabolic functions within the microbiome crucial for the degradation of pectin found in pepper pericarps. Carbohydrate metabolism, especially the galacturonic acid pathway, which provided important energy and carbon sources for microbial communities, significantly affected the degradation of pepper peeling [28]. To fully understand the important microbial communities and their enzymatic functions during pepper peeling, it is necessary to further study the degradation rules of pepper pericarp by using non-targeted metabolomics.

### 3.5. Metabolites Profiles of the Pepper Pericarp during the Retting Process

**Figure 4:**
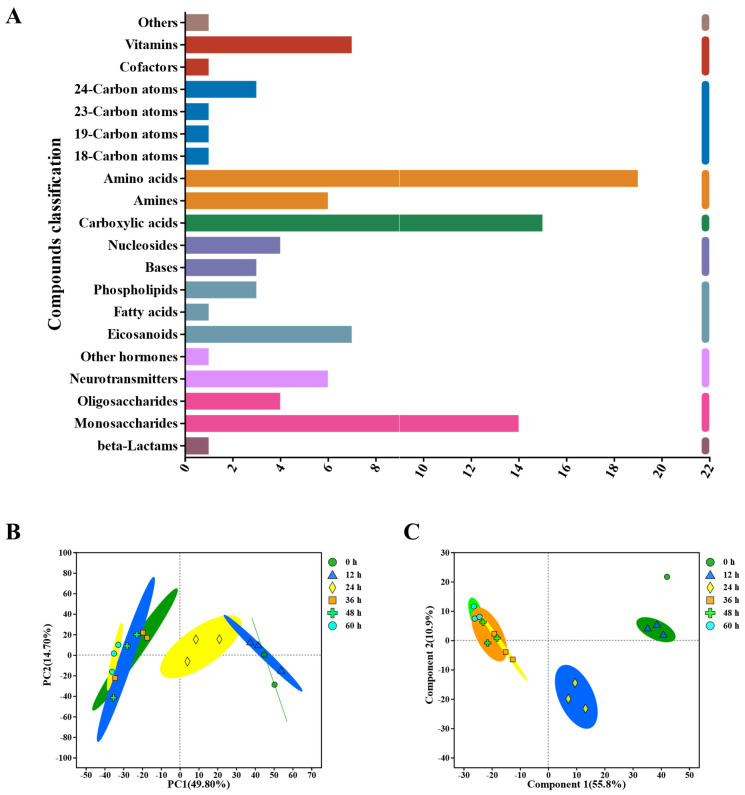
Untargeted Metabolomics evaluates metabolite profiles during the retting process, encompassing: (**A**) KEGG Compound Classification, (**B**) principal component analysis (PCA) of metabolites in the pepper pericarp, (**C**) Partial Least Squares Discriminant Analysis (PLS−DA) of metabolites in the pepper pericarp.

After identifying the key microbial communities during the peeling process, the metabolite profile of the pepper pericarp was investigated. The aim was to analyze the influence of the key microbiotas on the main components of the pepper pericarp. We used LC-MS and multivariate statistical methods to explore the dynamic changes in metabolic characteristics throughout the peeling process. The findings revealed a total of 1565 metabolites, including 24 peptides, 18 carbohydrates, 15 organic acids, 11 lipids, 8 vitamins and coenzymes, and 6 sterols (Figure 4A).

Using PCA analysis (Figure 4B), the metabolite profiles of the pepper pericarp were distinguished during the retting process. Further analysis showed that the first principal component (PC1) accounted for 49.8% of the changes in metabolite abundance, while the second principal component (PC2) contributed 14.7%. The PCA score chart clearly separated the pepper samples based on their metabolomic composition, illustrating different metabolic characteristics for each retting period.

Additionally, PLS-DA was employed to investigate the impact of time changes on the differences between pepper samples during the retting process (Figure 4C). The model demonstrated substantial predictive accuracy, with a coefficient of determination (R^2^) of 0.866 and a predictive validity (Q^2^) of 0.803. In this model, the principal components explained 55.87% and 10.9% of the total variance for the positive and negative ion combination patterns in PC1 and PC2, respectively. These results suggested that the metabolite profiles of the peel underwent significant changes throughout the retting process.

### 3.6. Metabolite Changes during the Degradation of Plant Cell Walls

**Figure 5:**
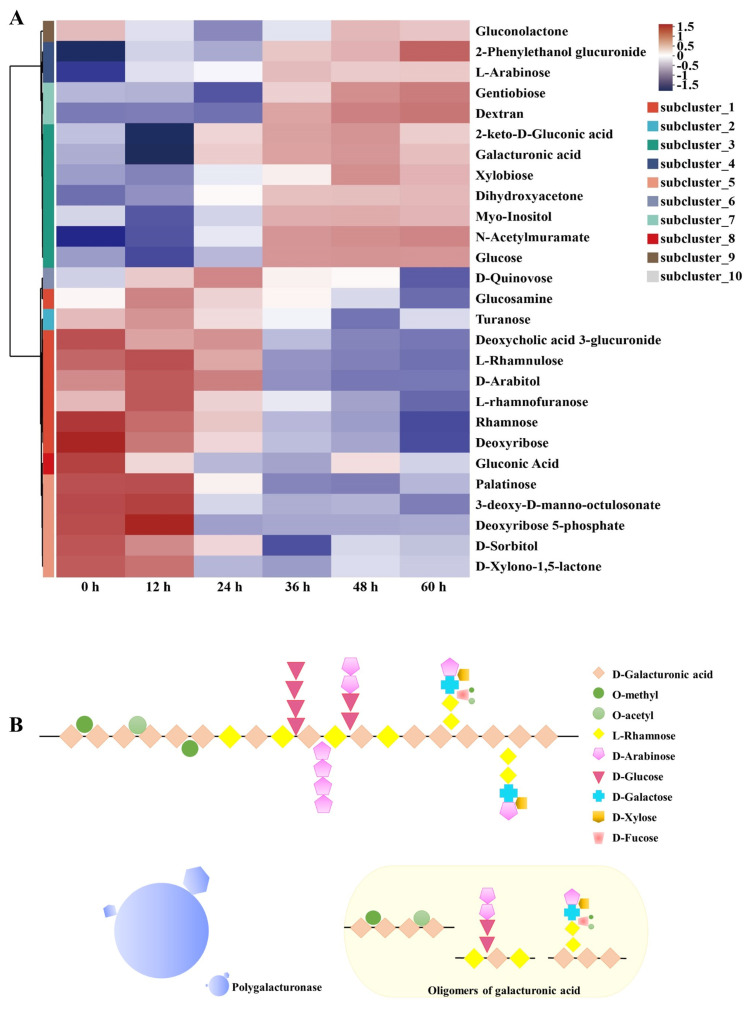
Clustering heatmap of differential carbohydrate metabolites (**A**). Schematic representation of the pectin degradation pathway (**B**).

The metabolites of pepper underwent significant changes, which were consistent with the enhancement of microbial metabolic functions. The microbial enzymes degraded pectin into monosaccharides, such as galacturonic acid, facilitating the separation of the pepper pericarp [29]. The metabolites produced from the degradation of pepper pericarp were transformed through microbial metabolic pathways. These transformations allowed the metabolites to serve as sources of nutrition for the microorganisms. Figure 5A illustrates the abundance of the carbohydrate metabolism set. The abundance of small molecular metabolites increased during the later stages of the retting process, such as galacturonic acid, glucose, and xylose. These trends in metabolite abundance were consistent with the dynamic changes in the microbial community. Heatmap analysis revealed the trends in abundance changes of the metabolites related to carbohydrate metabolism. The increase in galacturonic acid indicated the degradation of pectin. The increased abundance of glucose indicated the degradation of cellulose within the plant cell wall [30]. Hemicellulose, with its complex structure, was primarily composed of xylans, mannans, arabinans, and mixed-linkage β-glucans [31]. The increased abundance of l-arabinose and xylobiose indicated hemicellulose decomposition.

As shown in Figure 5B, polygalacturonase was capable of recognizing and cleaving the α-(1–4) glycosidic bonds between the polygalacturonic acid units within the pectin molecule, thereby degrading the main structure of pectin. Particularly, the changes in pectin metabolic products, including the rise in galacturonic acid and monosaccharides like glucose and xylose, further attest to the extent of pectin degradation [32]. The increase in pectinase activity was key to the efficient separation of the pepper pericarp, directly facilitating the disassembly of the pectin structure, thereby impacting the overall integrity of the pericarp and revealing the central role of pectinase in the peeling process [33].

### 3.7. Verification of Key Microbial− Encoded Enzymes in Accelerating Retting

**Figure 6:**
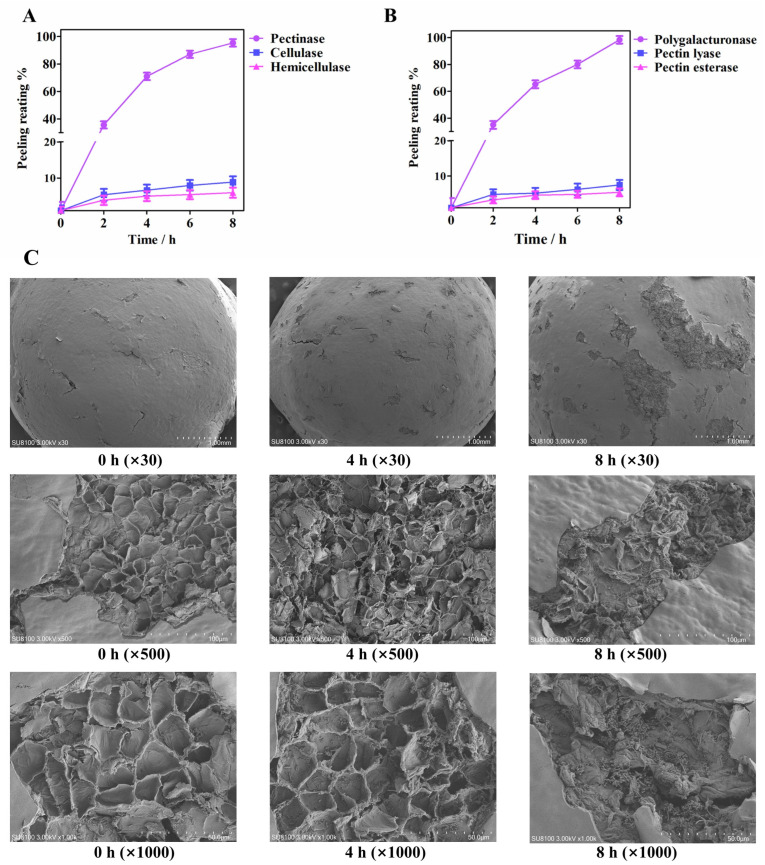
Pepper pericarp exfoliation verification of plant cell wall hydrolases: (**A**) Exfoliation experiments of pectinase, cellulase, and hemicellulase; (**B**) Pepper pericarp exfoliation experiments of polygalacturonase, pectin lyase, and pectin esterase; (**C**) Scanning electron microscope images were obtained of the pepper pericarp that was soaked in PG.

To validate the role of plant cell wall hydrolases, as predicted by key microbiota, in the pepper peeling process, we conducted a series of enzyme verification experiments, including those involving pectinase, cellulase, and hemicellulase. The results indicated that pectinase significantly accelerated the peeling process of pepper, while cellulase and hemicellulase had little effect (Figure 6A,B). Further experimentation with polygalacturonase, pectin lyase, and pectinesterase confirmed that only polygalacturonase could achieve complete peeling within 8 h, highlighting its important role in this process.

Figure 6C illustrates the microstructural changes in the pepper pericarp during the retting process after pretreatment with mechanical friction and exposure to the enzyme solution. Scanning electron microscopy revealed that at 0 h, cracks formed on the exocarp of the pepper, which were caused by mechanical friction pretreatment. The mesocarp within the cracks exhibited a net-like structure. After being soaked in the enzyme solution for 4 h, the cracks on the exocarp of the pepper widened, and the fracture appeared in the net-like structure. After being soaked in the enzyme solution for 8 h in the enzyme solution, the net-like structure had transformed into an obvious fragmented one. These observations revealed the important role of polygalacturonase in the peeling process of peppers, correlating with a decrease in pectin content and an accumulation of galacturonic acid, consistent with metabolomic findings. These results demonstrated that polygalacturonase could accelerate the pepper peeling process by degrading pectin. The decrease in pectin content and the accumulation of galacturonic acid were related to the activity of polygalacturonase. In the spontaneous soaking process, microorganisms degraded the pectin in the pepper pericarp through enzymatic action, and the accumulated monosaccharides promoted microbial growth through metabolic pathways. These results suggested a complex interaction between microbial community succession and pericarp structure decomposition through the action of enzymes.

## 4. Conclusions and Future Directions

Overall, the pectin content of the pepper pericarp decreased as a result of the activity of polygalacturonase produced by the microbiota, which effectively degrades the pectin during the retting process. *Prevotella*, *Lactococcus*, *Selenomonas*, *Candida*, and *Fusarium* were identified as the dominant microbial communities during the retting process. The correlation network elucidated the associations between the dominant microbiota and metabolic pathways, showcasing their essential roles in aiding the peeling process. Differential metabolite analysis identified galacturonic acid, which is a biomarker for pectin degradation, as exhibiting an increased trend in the pepper pericarp, suggesting a correlation between pectin degradation and an increase in the peeling rate. Subsequent experiments with key enzymes confirmed that polygalacturonase was primarily responsible for the effective breakdown of pectin, leading to the disintegration of the pepper pericarp’s cell wall and ultimately enabling the complete removal of the pepper pericarp.

This study has shown that the retting process involves a complex microbial community. However, an in-depth analysis of the enzyme systems produced by the microorganisms has not yet been conducted. To increase the controllability of the pepper peeling process, the development and implementation of a stable microbial community are essential. Therefore, future research could utilize proteomics to elucidate the changes in the enzyme system during the retting process and explore the variations in enzyme activity under different environmental conditions. Further identification of key microorganisms is needed, as well as an understanding of the expression levels of critical enzymes and the regulatory mechanisms of the microbial community. Based on this, the modification of key microorganisms could be carried out through biological methods such as DNA recombination. This step is considered essential to achieving a functionally stable microbial community. This process is important for the industrial application of this process and improving the efficiency and quality of white pepper production.

## Figures and Tables

**Table 1:** Dynamic changes of the key parameters in the retting process.

Retting Time (h)	0	12	24	36	48	60
pH	5.50 ± 0.07 c	5.62 ± 0.22 b	5.83 ± 0.20 a	5.92 ± 0.17 a	5.66 ± 0.15 a	5.50 ± 0.14 c
Peeling rate (%)	5.20 ± 0.43 a	16.08 ± 2.60 a	37.05 ± 5.17 b	67.62 ± 6.40 c	97.41 ± 0.73 d	99.67 ± 0.47 e
Pectin (mg/g)	24.18 ± 1.98 a	17.96 ± 2.05 b	16.86 ± 2.19 b	15.94 ± 2.58 b	9.46 ± 1.87 c	8.13 ± 1.98 c
Cellulose (mg/g)	102.61 ± 2.17 a	96.11 ± 2.41 b	89.82 ± 3.41 c	65.80 ± 3.32 d	61.15 ± 3.30 de	57.69 ± 3.47 e
Hemicellulose (mg/g)	35.96 ± 2.34 a	32.73 ± 2.53 a	28.72 ± 2.43 b	24.71 ± 2.46 c	24.22 ± 2.44 c	24.18 ± 2.36 c
Pectinase (mg/h/g)	3.36 ± 0.17 e	4.07 ± 0.20 d	5.78 ± 0.16 c	8.44 ± 0.17 b	8.34 ± 0.17 a	8.74 ± 0.09 b

Different lowercase letters (a–e) indicate significant differences in the value of the same factor between sampling time points, (*p* < 0.05). Values represent the mean ± standard deviation from three replicate experiments.

## Data Availability

The original contributions presented in the study are included in the article, further inquiries can be directed to the corresponding author.
